# Detections of Rabbit Hemorrhagic Disease Virus 2 (RHDV2) Following the 2020 Outbreak in Wild Lagomorphs across the Western United States

**DOI:** 10.3390/v16071106

**Published:** 2024-07-10

**Authors:** Jourdan M. Ringenberg, Kelsey Weir, Timothy Linder, Julianna Lenoch

**Affiliations:** National Wildlife Disease Program, Wildlife Services, Animal and Plant Health Inspection Service, U.S. Department of Agriculture, Fort Collins, CO 80521, USA; kelsey.weir@usda.gov (K.W.); timothy.j.linder@usda.gov (T.L.); julianna.b.lenoch@usda.gov (J.L.)

**Keywords:** RHDV2, lagomorph, wildlife, United States, rabbit, hare, lagoviruses (*Caliciviridae*), epidemiology

## Abstract

Rabbit hemorrhagic disease virus 2 (RHDV2) is a highly infectious, often fatal viral disease that affects both domestic and wild lagomorph species. In the United States (U.S.), the virus first was detected in wild lagomorph populations in the southwest in March 2020 and has continued to be detected in native North American lagomorph species over several years. The susceptibility of host species and exact mechanisms of environmental transmission across the U.S. landscape remain poorly understood. Our study aims to increase the understanding of RHDV2 in wild lagomorph populations by providing a history of detection. We present and summarize results from all RHDV2-suspect wild lagomorph morbidity and mortality samples submitted for diagnostic testing in the U.S. from March 2020 to March 2024. Samples were submitted from 916 wild lagomorphs across eight native North American species in 14 western states, of which 313 (34.2%) tested positive by RHDV2 RT-qPCR. Detections of RHDV2 in pygmy rabbits (*Brachylagus idahoensis*) and riparian brush rabbits (*Sylvilagus bachmani riparius*) suggest that the risk to threatened and endangered species warrants more attention. Continuing to investigate wild lagomorph morbidity and mortality events and tracking RHDV2 detections over time can help inform on disease epidemiology and wild lagomorph population trends.

## 1. Introduction

Rabbit hemorrhagic disease virus 2 (*Lagovirus europaeus* GI.2/RHDV2/b [1]; hereafter RHDV2) is a highly infectious, often fatal viral disease that affects both domestic and wild lagomorph species. Compared to classical rabbit hemorrhagic disease virus (*Lagovirus europaeus* GI.1/RHDV; hereafter RHDV), RHDV2 infects a broader range of species [2,3,4,5,6,7], can cross the species barrier and infect wild lagomorphs, and can cause mortality in kits as young as 11 days in addition to adult animals [8]. While initial research suggested that RHDV2 has a longer possible duration of disease than RHDV [6], recent investigations of RHDV2 suggest more virulent isolates are circulating that may have increased pathogenicity and cause a more rapid disease progression [9,10,11]. After first emerging in France in 2010 [12], RHDV2 rapidly spread to multiple countries throughout the subsequent decade, causing mortalities in both domestic and wild lagomorph populations. The first incursion of RHDV2 into the United States (U.S.) occurred in domestic rabbits (*Oryctolagus cuniculus*) in Ohio (OH) in 2018 and subsequently in Washington (WA) in 2019 and New York (NY) in 2020. In March 2020, the virus was detected for the first time in wild lagomorph populations in the southwestern U.S., has since swept across the western U.S., and has negatively affected several native North American lagomorph species.

Despite the U.S. outbreak entering its fifth year as of March 2024, the exact mechanisms of environmental transmission of RHDV2 across the U.S. landscape remain poorly understood [13]. Evidence has shown the virus can spread through contact (direct or indirect) with infected lagomorphs, contaminated materials or environments (e.g., forage, bedding), lagomorph carcasses, and wildlife vectors such as insects, raptors, and mammalian carnivores [14]. While the primary route of transmission is oral, other routes have been suggested, such as nasal, intravenous, intramuscular, and subcutaneous [2]. Previous research has shown that RHDV2 is particularly hardy in the environment, and infected carcasses can increase the viral load in the landscape [13], thus posing challenges to controlling disease spread. Given the rapid spread of RHDV2 across the globe and the presence of the virus in remote areas, human-mediated movement is suspected [15].

The number of known species susceptible to RHDV2 has expanded, yet there are still wild lagomorph species whose susceptibility to the virus is unknown, such as the American pika (*Ochotona princeps*). The expanded species range observed in the U.S. outbreak has raised concern for threatened and endangered (T&E) species. There are 19 extant, native wild lagomorph species whose geographic ranges fall entirely or partially within the U.S. [16] (Table 1). Of the native species, four are nationally classified as Endangered [17], whereas the International Union for Conservation of Nature (IUCN) Red List classifies only one species as Endangered [18], one Near Threatened [19], and three as Vulnerable [20,21,22]. Further, of species on the IUCN Red List classified as Least Concern, six list population trends as Unknown [23,24,25,26,27,28] and six list trends as Decreasing [29,30,31,32,33,34]. A better understanding of population dynamics for these species is necessary to detect population declines and ensure appropriate management action is taken.

Wild lagomorph population declines as a result of RHDV2 could impact trophic webs and ecosystems. For instance, in the U.S., several studies have identified *Sylvilagus* spp. as one of the most common prey options selected by breeding golden eagles (*Aquila chrysaetos*), and researchers have noted the impact of cottontail abundance on golden eagle mortality, reproduction, and habitat selection [35]. Many lagomorph species play significant roles in their respective ecosystems due to their ability to change vegetation structure and composition through seed dispersal and grazing [36]. Rabbit latrines impact soil fertility, facilitate plant growth and provide food resources for invertebrates, and warrens can provide refuge for other species. Cottontails and jackrabbits in the western U.S. are keystone prey species for several avian and mammalian predators [37], and lagomorph population declines can have deleterious impacts on predators that depend on lagomorphs as a primary food resource. Following the RHDV outbreak in China in 1984, declines were noted in T&E predator populations dependent on lagomorphs as a food resource [38]. Similarly, Monterroso et al. [39] evaluated the impact of the decline of European rabbits (*Oryctolagus cuniculus*) following the outbreak of RHDV2 in the Iberian Peninsula and noted severe negative impacts to lagomorph dependent predator populations: Iberian lynx (*Lynx pardinus*; Endangered [40]) and Spanish Imperial eagle (*Aquila adalberti*; Vulnerable [41]). Thus, RHDV2-caused population declines in wild lagomorphs could cause negative impacts on ecosystems.

The aim of this work is to report the state of RHDV2 in wild lagomorphs in the U.S. throughout the course of the southwestern U.S. outbreak. We tracked all U.S. RHDV2-suspect wild lagomorph morbidities and mortalities (M/M) submitted for diagnostic testing from March 2020 to March 2024. We present, review, and summarize the most comprehensive dataset of wild lagomorph samples submitted for RHDV2 testing in the U.S. Our objective is to increase the global body of knowledge on RHDV2 by providing a history of detection to better understand its impact on wild lagomorphs.

## 2. Materials and Methods

Wild lagomorphs with a suspicion of RHDV2 were collected opportunistically across the U.S. between March 2020 and March 2024 as part of M/M events by state wildlife and agricultural agencies, federal government agencies, Tribal Nations, non-government organizations, rehabilitation facilities, and private citizens. The most prevalent field sign noted that indicated suspicion of RHDV2 was blood around the nares and/or mouth. Many lagomorphs were submitted as part of mortality events where animals were found dead with no apparent signs of disease or cause of death. Lagomorphs often were found in areas where wild or domestic die-offs previously had been reported. Signs of disease were reported upon sample submission to the lab and any remarkable lesions were noted during necropsy. Lesions suggestive of RHDV2 included congested and friable liver; liver necrosis and autolysis; pulmonary congestion and hemorrhage; and autolytic blood in abdominal and thoracic cavities.

All state and federal agencies and Native nations with regulatory authority over wild lagomorphs were eligible to submit diagnostic samples [42]. Precise collection methodology may have differed depending on the state and collecting entity; thus, we are unable to report on specific state methodologies for collecting RHDV2-suspect samples. Rather, we report the guidance for collecting and shipping RHDV2-suspect samples issued in August 2020 to all state and federal agencies by the U.S. Geological Survey National Wildlife Health Center (NWHC; Madison, Wisconsin, U.S.) [42]. All North American wild lagomorph species that were part of M/M events were eligible for RHDV2 diagnostic testing. Samples were accepted from all species in any state and county only until the virus was detected in a given species in that county. Subsequent submissions from that county would only be accepted for testing if they represented a new species with no documented detections in that county. Lagomorph species were identified on a morphological basis by the submitting wildlife biologists, veterinarians, or rehabilitators and confirmed at the lab during evaluation [43]. All unknown species were classified as unidentified by genus. The type of sample submitted was dependent on the collecting entity; most samples submitted were either whole carcasses or liver, as liver tissue is the current gold standard for RHDV2 testing [44]. Less common sample types submitted were bone marrow, ear tissue, kidney, spleen, and rectal swabs. Samples were to be chilled or frozen immediately following collection or death and shipped to a diagnostic lab within 24–36 h [42].

All wild lagomorph RHDV2-suspect samples were either sent directly to a national or state lab approved for RHDV2 diagnostic testing or forwarded from a non-approved lab. Labs approved for RHDV2 testing include the U.S. Department of Agriculture (USDA) National Veterinary Services Laboratories Foreign Animal Disease Diagnostic Laboratory (FADDL; Plum Island, NY, U.S.), the NWHC, the Southeastern Cooperative Wildlife Disease Study (SCWDS; Athens, Georgia, U.S.), and several state wildlife labs. All samples collected prior to July 2020 were submitted to FADDL; in July 2020, the USDA designated the NWHC and SCWDS as approved labs for RHDV2 diagnostic testing. Also at this time, RHDV2 testing protocols were provided to state wildlife agencies, and testing capabilities were expanded to include several state diagnostic labs.

Samples first submitted to a non-approved RHDV2 lab were necropsied and an attempt was made to determine cause of death. Per protocol [42], the NWHC conducted comprehensive cause-of-death assessments for all large (greater than five animals) M/M events, whereas samples from small M/M events were limited to RHDV2 virology testing only, unless they were T&E species. Cause-of-death assessment protocols may have differed depending on the lab. Any sample with lesions indicative of RHDV2 or an undetermined cause of death were forwarded to a RHDV2-approved lab. Samples from a new state, county, or species that tested positive for RHDV2 at the NWHC, SCWDS, or an approved state lab were classified as “preliminary positive” and were forwarded to FADDL for confirmatory testing.

Once received at an RHDV2-approved diagnostic lab, liver sections were taken from each animal and homogenized in a phosphate-buffered saline. The liver homogenates were vortexed and centrifuged; nucleic acid was then extracted from the supernatant and eluted in RNase-free water [45]. The eluted samples were tested for the presence of RHDV2 nucleic acids using a real-time TaqMan-probe-based reverse-transcription polymerase-chain reaction (RT-qPCR) targeting the *vp60* gene, as previously described [45]. Cycle threshold values less than 35 were interpreted as positive.

All samples were tested promptly upon arrival at diagnostic laboratories, but variation in shipping times may have occurred, and as such, the date a sample was tested may have been significantly later than the date the animal died or was collected. Collection dates were not available for all samples, and thus, all dates reported are based on the date samples were tested. All detections are reported at the U.S. County level due to the collection of many animals from private residences and by non-government entities.

The USDA/National Wildlife Disease Program (NWDP) began assisting USDA/Veterinary Services (VS) with the tracking of wild lagomorph RHDV2 detections in May 2020 and by mid-2021, had taken sole responsibility for this role. A standardized reporting protocol was collaboratively developed by VS, the NWDP, and the NWHC for both wild and domestic detections. For wild lagomorphs, the submitting agencies within each state were expected to contact the NWDP to report all RHDV2-suspect submissions and diagnostic results, and the NWDP reported all results to the appropriate state personnel.

## 3. Results

A total of 916 wild lagomorphs from RHDV2-suspect M/M events were tested between March 2020 and March 2024 (Table S1). Samples tested included those from three genera of wild lagomorphs: 8 *Brachylagus*, 127 *Lepus*, and 781 *Sylvilagus* (Table 2). Samples originated from 42 U.S. states and were submitted to and tested by 16 federal and state labs approved for RHDV2 testing.

**Figure 1:**
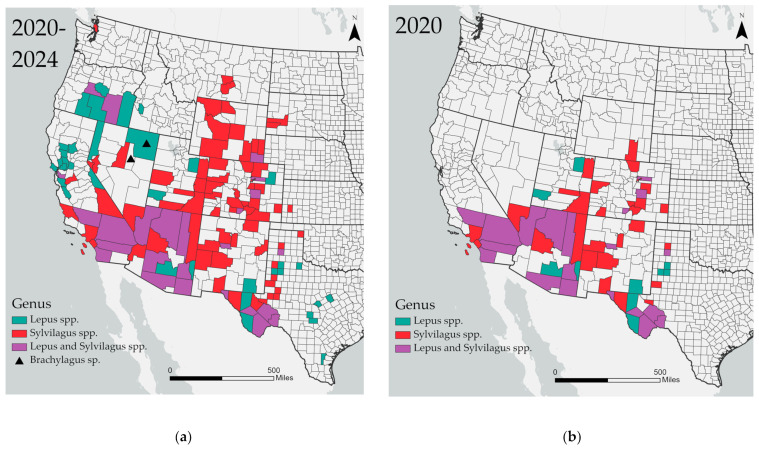

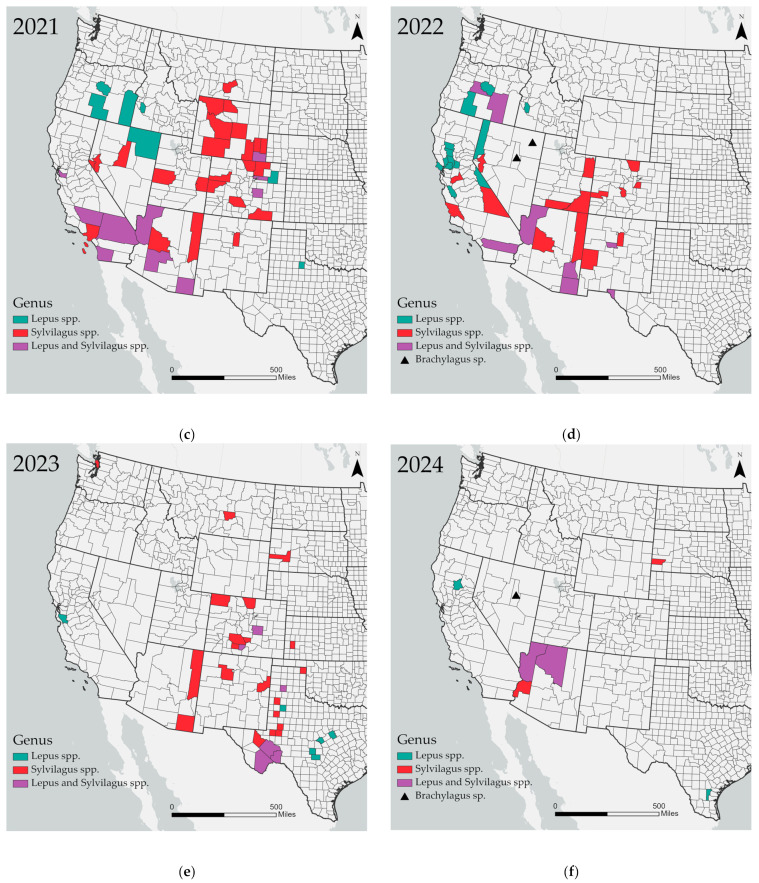
Detections of RHDV2 by U.S. County and genus during: (**a**) entire outbreak period, 2020–2024; (**b**) 2020; (**c**) 2021; (**d**) 2022; (**e**) 2023; (**f**) 2024. Map (**a**) is the composite of maps (**b**–**f**). More than one detection per genus may be represented in each positive county. Each mapped year represents a 12-month calendar year except 2020 (Mar–Dec) and 2024 (Jan–Mar).

A total of 313 (34.2%) lagomorphs tested positive for RHDV2 by RT-qPCR in eight native North American lagomorph species (Table 2) across 14 western states (Figure 1). Of the species nationally classified as endangered, RHDV2 was detected in both pygmy rabbits (*Brachylagus idahoensis*) and riparian brush rabbits (*Sylvilagus bachmani riparius*). Samples also were submitted for marsh rabbits (*Sylvilagus palustris*) and New England cottontails (*Sylvilagus transitionalis*); however, no detections were reported in either species.

**Figure 2:**
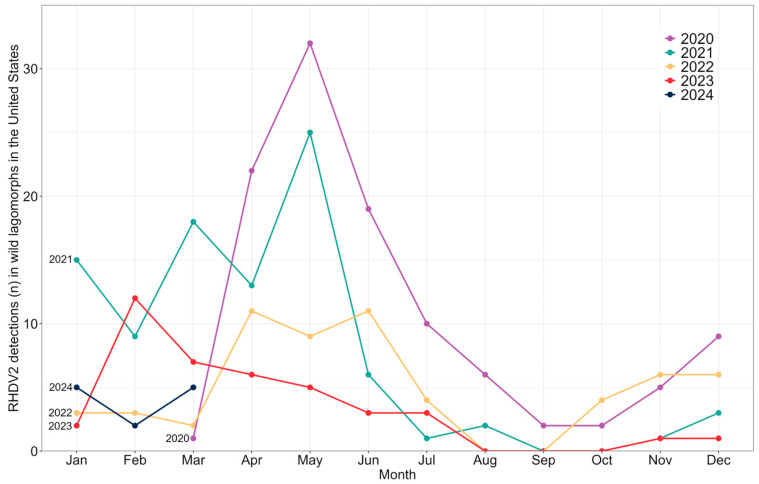
Monthly U.S. RHDV2 detections by year in wild lagomorphs from March 2020 to March 2024. Each calendar year is represented as a uniquely colored line.

Samples were tested throughout the four-year period, with an increase in 2021 followed by a decreasing trend observed in the subsequent years: 2020 (254), 2021 (274), 2022 (193), 2023 (116), 2024 (79). While the total number tested from January to March in 2024 is greater than years 2022 and 2023 during the same three-month periods, 57 of those samples were from one state (North Carolina) with zero reported detections. Detections of RHDV2 generally appeared to increase during winter (Jan–Mar) and spring (Apr–Jun), and decrease during summer (Jul–Sep) and fall (Oct–Dec) seasons (Figure 2).

## 4. Discussion

In this study, we present data from the first four years (March 2020–March 2024) of the RHDV2 outbreak in the U.S. A total of 13 native wild lagomorph species were tested diagnostically for RHDV2, and detections were reported in eight of those species (Table 2). The highest number of tests were conducted on black-tailed jackrabbits, desert cottontail rabbits, and eastern cottontail rabbits, all of which often occupy anthropogenically modified landscapes. Desert cottontails do well in exurban areas, while eastern cottontails and black-tailed jackrabbits are habitat generalists and adapt well to many habitat types [16,46]. The proximity of these species to human populations makes it more likely sick or dead individuals will be found in the landscape before being removed by predators or scavengers, versus more elusive or habitat specialist lagomorph species whose mortalities may go unnoticed by humans.

Although the mortality rate of RHDV2 in native wild lagomorph species in the U.S. is unknown, evidence has shown that some individuals do survive exposure [47], and the epidemiology may be species-dependent [44]. Disease outbreaks may have greater implications for T&E lagomorph species already facing conservation challenges. With detections in both pygmy and riparian brush rabbits, the risk RHDV2 poses to T&E species is concerning. Conservation strategies, management plans, or recovery plans exist for the T&E New England cottontail [48], Columbia Basin pygmy rabbit [49], riparian brush rabbit [50], and Lower Keys marsh rabbit [51] across all or part of their respective ranges. Continuing to monitor these populations will contribute insights on how RHDV2 presents in these species and provide critical information on mortality rates [52]. Population monitoring is also particularly important for species with unknown and declining population trends (Table 1) so that population declines are detected early, and appropriate management action can be taken. Performing serology on apparently healthy wild lagomorphs or those that test negative for RHDV2 by RT-qPCR can provide valuable information on evidence of exposure, susceptibility, and survival rates. Future investigations should consider conducting serological surveys of T&E species, especially in those species that were submitted for RHDV2 testing but in which the virus was not detected (i.e., New England cottontail and Lower Keys marsh rabbit). Results from the experimental inoculation of domestic and eastern cottontail rabbits with RHDV2 suggest eastern cottontails could be less susceptible to lethal infection [47]. Understanding differences in susceptibility between species is imperative to better manage the disease risks and reduce the impact on wild populations.

Detections of RHDV2 generally decreased during the summer (Jul–Sep) and fall (Oct–Dec), and increased during the winter (Jan–Mar) and spring (Apr–Jun) seasons (Figure 2). Researchers in Portugal observed a cyclical pattern in Iberian rabbits (*Oryctolagus cuniculus algirus*), with higher RHDV2 mortality rates associated with the coldest months (January and February), which may be correlated to the rabbit breeding season that begins in early winter and continues into spring [15]. Sun et al. [53] identified spring peaks of RHDV2 outbreaks in wild rabbit populations in the northern hemisphere, a pattern likely attributable to reproductive cycles. Investigations of RHDV have shown similar cyclical patterns, with high mortality rates occurring in colder months in conjunction with the annual influx of susceptible young rabbits [54]. Serology investigations in Australia suggest that the increase in young, naïve rabbits during the breeding season can cause high rates of RHDV2 viral shedding and mortality, effectively contaminating the environment with infected carcasses that further assist in virus transmission [55]. Cottontail species breed as early as February [56,57], whereas the breeding season for black-tailed jackrabbits is variable and could begin as early as December [16]. The cyclical pattern of RHDV2 in the U.S. could correlate to lagomorph breeding behavior that involves increased contact rates between adults and results in an annual influx of susceptible young animals. Continuing to investigate wild lagomorph M/M events and tracking RHDV2 detections over time can help inform on seasonal trends.

Methodological limitations may have introduced biases into our data. Restricting collection methodology to only M/M individuals removed the opportunity to detect and monitor RHDV2 in apparently healthy lagomorphs. Conducting seroepidemiological surveys could provide valuable data on evidence of exposure and survival rates in these individuals. Our results showed the total number of samples tested began decreasing in 2021; however, this may not be representative of population trends or RHDV2 in the landscape as testing only the first sample in each county and species meant the eligible population decreased over time. Further, data were limited to what was reported to the NWDP and may not include all samples tested across the country. These limiting factors may have resulted in an underestimation of the virus in the landscape and should be considered when designing future research.

The limitations of our data prevented the ability to conduct sex or age analyses. While standardized forms submitted to diagnostic testing labs alongside RHDV2-suspected samples contained the option to include sex and age information, these were recorded only for 40% of samples. Ensuring these data are included when submitting samples for testing is imperative in understanding if and how these variables factor into disease risk and viral transmission. Additionally, because no detailed numerator or denominator data were available, the actual prevalence and mortality rates could not be calculated from the reported dataset.

There are many gaps in knowledge regarding the transmission of RHDV2 in wild lagomorphs in the U.S. and the role of interspecies dynamics. Detections in wild lagomorphs thus far have been restricted to the western U.S., despite domestic rabbit detections spanning into eastern states. Human-mediated transmission of fomites is a suspected cause for the geographic jumps seen in domestic rabbit cases. Human interaction with domestic rabbit populations is common and provides one potential explanation as to why wild lagomorph detections have not followed a similar geographic trend. Regardless, the presence of RHDV2 in domestic rabbits in the eastern U.S. does pose a threat to wild populations, especially in areas with inadequate containment facilities and improper disposal of contaminated bedding, feed, or carcasses [13]. Genetic sequencing performed on 12 RHDV2 positive samples from California found similarities between viruses obtained from sympatric domestic and wild lagomorphs, suggesting interspecies transmission [58]. There is likely more complexity in virus transmission in areas with sympatric lagomorphs [13], and shared infections may circulate between populations [2]. Research in Australia and Europe demonstrated similar virus dynamics and suggested that the detection of RHDV2 in European brown hares (*Lepus europaeus*) was the result of a spillover event from rabbits [6,59]. Implementing sound biosecurity practices when working with domestic lagomorphs is imperative to reduce virus transmission risk to wild lagomorphs.

The extent to which other mechanisms of viral transmission impact RHDV2 ecology in the U.S is uncertain. Carcasses in the landscape may play a large part in the maintenance of RHDV2 in wild lagomorphs [2], and insect, bird, and other mammalian vectors may facilitate virus transmission [58]. While not thought to be able to replicate the virus, species scavenging and preying upon lagomorphs may excrete the virus in fecal material or carry it on claws, feet, and regurgitated materials [60]. Investigations in Italy of European brown hare syndrome, a *Lagovirus* classified with RHDV, have detected viral strains in wolf (*Canis lupus*) fecal material [61] and the intestinal contents of a red fox (*Vulpes vulpes*) [62]. RHDV2 has been reported in other mammal species (e.g., voles [*Microtus*], mice [*Mus*], badgers [*Meles*]), but it is uncertain whether these species act as permissive hosts or simply mechanical vectors [63,64,65]. Further, the effect survivors have on the transmission cycle is unknown and may be species dependent [13]. Experimental inoculation demonstrated that eastern cottontails are susceptible to RHDV2 and can shed viral RNA, yet their mortality rates were lower than domestic rabbits, suggesting eastern cottontails could become infected and mechanically transmit the virus across the landscape [47,58]. Given the widespread distribution of eastern cottontails in the U.S., including their synanthropic behavior and sympatric nature with other wild lagomorphs (e.g., New England cottontail, swamp rabbit), monitoring populations may be necessary to better understand disease transmission and protect more sensitive species.

Climate is another potential factor influencing RHDV2 transmission and rates of infection. Arid and semi-arid regions span much of the western U.S., whereas regions in the eastern U.S. are more humid. It has been suggested that RHDV can cause more significant population declines in arid and semi-arid regions [66]. After its introduction as a biocontrol agent in Australia, a greater reduction in rabbit populations was seen in more arid regions [14]. This pattern could be influenced by lagomorph population dynamics (i.e., more reproductive success in areas with greater rainfall) [67] or the seasonal abundance and movement of potential RHDV vectors [68]. The geographical pattern of RHDV2 detections in the U.S. provides supporting evidence to this theory, but more research is needed to better understand the role climate plays in RHDV2 epidemiology.

Landscape characteristics and time of year could impact disease spread and contribute to the western restriction of RHDV2. Habitat fragmentation due to anthropogenic development may influence lagomorph movement and subsequent virus transmission. Apart from Texas (TX) and Kansas, RHDV2 cases in wild lagomorphs do not appear to geographically traverse across the Great Plains, a region with significant cropland expansion and decrease in native grassland [69]. Research on eastern cottontail rabbits in Nebraska, U.S., suggests that they may select for grassland habitats and avoid using crop fields when crop height is insufficient to provide adequate refuge [70]. During the winter and spring months, some lagomorphs may be isolated in small, native habitat fragments with little opportunity for safe movement to other fragments. Thus, landscape quality and composition may influence the environmental transmission of RHDV2 and should be considered when evaluating populations.

Although fragmented landscapes could help prevent viral transmission, they may exacerbate the deleterious effects of disease outbreaks on isolated metapopulations of lagomorphs [2]. Evidence has shown that habitat patch size is negatively correlated to extinction rates [71]. An RHDV2 outbreak could lead to prolonged extirpation of a small, isolated metapopulation, and T&E lagomorph species have been shown to be particularly susceptible to the combined deleterious effects of RHDV2 and habitat fragmentation. Declines in pygmy rabbit and Lower Keys marsh rabbit populations across their respective ranges have been connected to increases in movement-restricting landscape barriers attributable to habitat fragmentation [72,73]. After comparing pygmy rabbit numbers pre- and post-RHDV2 detections, Crowell et al. [52] concluded that RHDV2 significantly impacted the Nevada, U.S., population.

Future investigations of RHDV2 outbreaks should consider molecular epidemiology to provide genetic insights into geo-temporal distribution and monitor for recombination events. Whole genome sequencing of wild and domestic lagomorph isolates from the southwestern outbreak indicates a distinct incursion event into the area separate from the previous domestic detections in OH 2018, WA 2019, and NY 2020. Sequencing was conducted at FADDL of wild (*n* = 1) and domestic (*n* = 4) lagomorphs from Arizona (AZ), Colorado (CO), New Mexico (NM), and TX [74]. Genomes from AZ, NM, and TX showed a single genetic cluster that was distinct from the OH 2018, WA 2019, and NY 2020 domestic rabbit sequences. Researchers in California sequenced 12 lagomorph isolates (wild (*n* = 6), and domestic (*n* = 6)) and found all 12 to be closely related to each other and similar to the sequences from AZ, NM, and TX, supporting the theory of a single incursion event into the region [58].

Vaccinating wild lagomorphs comes with logistical challenges but may be a vital tool for the conservation of T&E populations. A recombinant subunit protein RHDV2 vaccine (developed by Medgene Labs [Brookings, South Dakota, U.S.]) is available for use by licensed veterinarians in 44 U.S. states [75], and challenge studies showed a 0% mortality rate for vaccinated domestic rabbits (*Oryctolagus cuniculus*) [76]. While vaccinating wild lagomorph populations is not feasible in many cases, spatial modelling of RHDV2 in a California riparian brush rabbit population predicted that a mere 30% vaccination rate would yield conservation benefits and prevent population decline. Approximately 1.5 years after a vaccine program was initiated in this population, RHDV2 was detected and caused mortalities in unvaccinated riparian brush rabbits. Frequent vaccination may be necessary due to short lagomorph lifespans and high population turnover [13], but long-term vaccination is costly and logistically challenging. Targeting small at-risk populations is more realistic than widespread vaccination efforts.

Sound biosecurity practices are pertinent to reduce human-mediated spread of RHDV2 to and between wild and domestic lagomorph populations. General guidance by the USDA suggests the following: (1) wear site-specific clothing and personal protective equipment (PPE) when handling lagomorphs and clean and disinfect boots, PPE, and vehicles, using a 10% bleach or 10% sodium hydroxide solution, when moving between sites; (2) use new disposable gloves for each animal when working with multiple lagomorphs; (3) contact the appropriate state or federal animal health official for guidance on proper carcass disposal; (4) refrain from contact with domestic rabbits before or after handling wild lagomorphs; and (5) do not release domestic rabbits into the wild or bring wild lagomorphs into a domestic population [77].

Management efforts should address the conservation needs of specific species and populations, but broad steps can be taken to protect lagomorph populations and increase understanding of RHDV2 dynamics. Land management activities and environmental impact assessments should consider the habitat available to wild lagomorphs as landscape connectivity is imperative to mitigate disease impacts [52]. Conservation plans should take measures to improve rates of survival during increased outbreak periods, such as winter and spring, the result of which could allow animals to seroconvert naturally and build immunity [15]. Conducting active surveillance in targeted areas through systematic sample collection and analysis could inform on disease prevalence and evolution, genetic resistance, and appropriate management actions. As recombination may be one mechanism of novel pathogenic lagovirus emergence, it is likely that RHDV2 may continue to evolve over time, stressing the need for ongoing monitoring of wild lagomorph populations.

## 5. Conclusions

The RHDV2 outbreak in wild lagomorphs in the U.S. has affected several native species across the western part of the country. Detections in the nationally endangered pygmy and riparian brush rabbits raise concern for threatened and endangered populations, which may warrant more attention. Vaccinating wild lagomorphs is an optional management action but likely is only feasible in small T&E populations. Detections appear to be higher during the winter and spring months, potentially correlating to lagomorph breeding behavior. Despite domestic rabbit detections throughout the eastern U.S., RHDV2 epidemiology in wild lagomorphs has not followed the same trend. The exact mechanisms of transmission across the landscape are speculative, and evaluating landscape characteristics could provide additional information on viral transmission. Continuing to investigate wild lagomorph morbidity and mortality events and tracking RHDV2 detections over time can help inform on disease epidemiology and wild lagomorph population trends.

## Figures and Tables

**Table 1:** International Union for Conservation of Nature (IUCN) Red List and United States Fish and Wildlife Service (USFWS) national-level assessment classifications of all native wild lagomorph species with geographic ranges entirely or partially located within the United States [16].

Common Name	Scientific Name	IUCN Red List Classification (Population Trend)	USFWS Classification
Collared pika	*Ochotona collaris*	LC ^1^ (Unknown)	NL ^5^
American pika	*Ochotona princeps*	LC (Decreasing)	NL
Pygmy rabbit	*Brachylagus idahoensis*	LC (Unknown)	EN— Columbia Basin DPS ^6^
Swamp rabbit	*Sylvilagus aquaticus*	LC (Decreasing)	NL
Desert cottontail	*Sylvilagus audubonii*	LC (Decreasing)	NL
Brush rabbit	*Sylvilagus bachmani*	LC (Stable)	EN— *S. b. riparius*
Manzano mountain cottontail	*Sylvilagus cognatus*	EN ^2^ (Unknown)	NL
Eastern cottontail	*Sylvilagus floridanus*	LC (Unknown)	NL
Mountain cottontail	*Sylvilagus nuttallii*	LC (Decreasing)	NL
Appalachian cottontail	*Sylvilagus obscurus*	NT ^3^ (Decreasing)	NL
Marsh rabbit	*Sylvilagus palustris*	LC (Unknown)	EN— *S. p. hefneri*
Davis Mountains cottontail	*Sylvilagus robustus*	VU ^4^ (Decreasing)	NL
New England cottontail	*Sylvilagus transitionalis*	VU (Decreasing)	EN
Antelope jackrabbit	*Lepus alleni*	LC (Unknown)	NL
Snowshoe hare	*Lepus americanus*	LC (Stable)	NL
Black-tailed jackrabbit	*Lepus californicus*	LC (Decreasing)	NL
White-sided jackrabbit	*Lepus callotis*	VU (Decreasing)	NL
Alaskan hare	*Lepus othus*	LC (Unknown)	NL
White-tailed jackrabbit	*Lepus townsendii*	LC (Decreasing)	NL

^1^ Least concern; ^2^ Endangered; ^3^ Near threatened; ^4^ Vulnerable; ^5^ Not listed; ^6^ Distinct population segment.

**Table 2:** Wild lagomorphs tested for RHDV2 by RT-qPCR in the U.S. between March 2020 and March 2024.

Genus	Species	Tested ^1^	Positives
*Brachylagus*	Pygmy rabbit	**8**	**7**
*Lepus*	Antelope jackrabbit	3	3
	Black-tailed jackrabbit	106	77
	Snowshoe hare	6	0
	White-tailed jackrabbit	3	0
	*Lepus* (unidentified)	9	8
	**Total *Lepus***	**127**	**88**
*Sylvilagus*	Brush rabbit	35	1
	Desert cottontail	236	121
	Eastern cottontail	260	9
	Marsh rabbit	11	0
	Mountain cottontail	15	6
	New England cottontail	4	0
	Riparian brush rabbit	28	4
	Swamp rabbit	11	0
	*Sylvilagus* (unidentified)	181	77
	**Total *Sylvilagus***	**781**	**218**
	**All species**	**916**	**313**

^1^ All lagomorphs tested were from RHDV2-suspect mortalities.

## Data Availability

The original contributions presented in the study are included in the Supplementary Material (Table S1); further inquiries can be directed to the corresponding author.

## Supplementary Materials

The following supporting information can be downloaded at: https://www.mdpi.com/article/10.3390/v16071106/s1. Table S1: Number of wild lagomorph species submitted for rabbit hemorrhagic disease virus 2 diagnostic testing with the first date of detection listed for each species, state, and county, where applicable.
