# Supplementary material for: Detections of Rabbit Hemorrhagic Disease Virus 2 (RHDV2) Following the 2020 Outbreak in Wild Lagomorphs across the Western United States
Source: Viruses. 2024 Jul 10;16(7):1106. doi: 10.3390/v16071106 (PMC11281353; doi:10.3390/v16071106)
Supplement: Supplementary file 1 [file viruses-16-01106-s001.zip › viruses-3073160-supplementary/ringenberg et al_rhdv2 in the US_supplemental table.pdf]

Table S1. Number of wild lagomorph species submitted for rabbit hemorrhagic disease virus 2 diagnostic testing with the first date of detection listed for each species, state, and county, where applicable.

Species	State	County	Detected	Not Detected	Date of First Detection
Antelope jackrabbit	Arizona	Pima	3	0	2020-05-01
Black-tailed jackrabbit	Arizona	Cochise	3	3	2020-04-08
		Coconino	2	0	2020-05-15
		Graham	1	0	2020-04-23
		Greenlee	1	0	2020-04-17
		Maricopa	0	2	N/A
		Mohave	1	0	2021-03-15
		Navajo	1	0	2020-05-01
		Pima	1	0	2020-03-31
		Pinal	1	0	2020-06-12
	California	Alameda	1	0	2021-06-24
		Butte	1	0	2024-02-23
		Colusa	1	0	2022-11-28
		Contra Costa	0	3	N/A
		El Dorado	0	1	N/A
		Glenn	2	0	2022-11-28
		Humboldt	0	1	N/A
		Kern	4	0	2020-12-01
		Los Angeles	0	1	N/A
		Marin	3	5	2022-10-07
		Mendocino	0	1	N/A
		Mono	1	0	2022-07-15
		Monterey	0	1	N/A
		Napa	1	0	2022-11-18
		Placer	0	1	N/A
		Riverside	1	0	2020-05-11
		Sacramento	1	1	2022-12-28
		San Benito	1	0	2022-01-05
		San Bernardino	2	0	2020-06-05
		San Diego	2	1	2021-02-10
		San Luis Obispo	0	1	N/A
		San Mateo	0	1	N/A
		San Ramon	0	1	N/A

		Santa Clara	1	1	2023-02-08
		Solano	1	1	2022-10-07
		Yolo	2	0	2022-10-17
	Colorado	Adams	2	0	2020-06-11
		El Paso	1	0	2020-05-21
	Idaho	Ada	4	0	2021-03-25
	New Mexico	Bernalillo	1	0	2020-05-01
		Eddy	1	0	2020-04-01
	Nevada	Clark	0	2	N/A
		Douglas	0	1	N/A
		Elko	2	0	2021-05-03
		Washoe	1	0	2022-05-23
	Oregon	Crook	2	0	2021-12-16
		Deschutes	1	1	2022-12-07
		Harney	1	1	2022-02-04
		Klamath	1	0	2022-04-28
		Lake	1	0	2021-06-08
		Malheur	1	0	2021-12-16
	Texas	Bosque	2	0	2023-03-09
		Brewster	2	0	2020-05-29
		Cottle	1	0	2021-04-06
		Culberson	1	0	2020-06-12
		Hale	1	0	2020-05-19
		Hockley	1	0	2020-05-08
		Jeff Davis	1	0	2020-05-07
		Jim Wells	1	0	2024-03-18
		Lubbock	2	0	2020-04-15
		Mills	1	0	2023-02-24
		Pecos	1	0	2020-05-07
		Presidio	1	1	2020-05-21
		Randall	1	0	2020-05-01
		Terrell	1	0	2020-04-29
	Utah	Iron	2	0	2020-11-10
	Wyoming	Laramie	3	0	2021-01-27
Brush rabbit	California	Contra Costa	0	2	N/A
		Los Angeles	1	0	2021-06-18
		Orange	0	1	N/A
		Placer	0	2	N/A
		San Diego	0	1	N/A

		San Joaquin	0	3	N/A
		San Mateo	0	2	N/A
		Santa Barbara	0	2	N/A
		Santa Clara	0	2	N/A
		Sonoma	0	1	N/A
		Ventura	0	1	N/A
		Yolo	0	1	N/A
	Oregon	Benton	0	1	N/A
		Clackamas	0	4	N/A
		Columbia	0	2	N/A
		Linn	0	1	N/A
		Marion	0	1	N/A
		Multnomah	0	1	N/A
		Tillamook	0	1	N/A
		Washington	0	5	N/A
Desert cottontail	Arizona	Apache	6	0	2021-01-15
		Cochise	14	0	2020-04-08
		Coconino	2	0	2020-06-19
		Graham	1	0	2022-05-20
		La Paz	1	1	2024-01-22
		Maricopa	0	7	N/A
		Mohave	9	1	2020-06-12
		Navajo	1	0	2020-11-20
		Pima	3	2	2020-05-01
		Yavapai	10	1	2020-05-22
	California	Alameda	1	1	2021-11-12
		Amador	0	1	N/A
		Contra Costa	0	3	N/A
		El Dorado	0	1	N/A
		Imperial	0	1	N/A
		Inyo	1	0	2022-07-15
		Kern	3	5	2020-11-24
		Los Angeles	4	8	2020-07-27
		Merced	0	8	N/A
		Orange	1	2	2020-06-22
		Placer	0	1	N/A
		Riverside	3	8	2022-05-16

		Sacramento	0	3	N/A
		San Bernardino	4	2	2020-06-12
		San Diego	9	18	2020-06-12
		San Francisco	0	1	N/A
		San Joaquin	0	8	2024-02-26
		San Luis Obispo	1	0	2022-06-14
		Santa Barbara	0	2	N/A
		Stanislaus	2	9	2022-05-25
		Turlock	0	1	N/A
		Ventura	0	14	N/A
		Yolo	0	1	N/A
	New Mexico	Catron	2	0	2020-06-05
		Cibola	1	0	2020-06-05
		Colfax	1	0	2020-06-05
		Dona Ana	5	0	2020-04-02
		Quay	1	0	2023-04-17
		Santa Fe	5	0	2020-04-27
		Socorro	1	0	2020-07-17
	Nevada	Clark	2	1	2020-06-22
		Douglas	1	0	2021-05-25
		Lander	1	0	2021-04-06
		Lincoln	0	2	N/A
		Lyon	4	0	2021-05-10
		Washoe	0	2	N/A
	Texas	Brewster	1	0	2023-02-07
		El Paso	1	0	2020-04-27
		Gaines	1	0	2020-05-22
		Hudspeth	1	0	2020-04-17
		Lamb	1	0	2023-03-30
		Lynn	0	1	N/A
		Pecos	1	0	2020-04-27
		Randall	0	1	N/A
	Utah	Kane	1	0	2022-07-11
		San Juan	1	0	2022-04-28
		Uintah	1	0	2022-04-04
	Wyoming	Albany	6	0	2021-01-27
		Bighorn	1	0	2021-05-19

		Fremont	1	0	2021-05-11
		Goshen	0	1	N/A
		Laramie	2	2	2021-02-12
		Natrona	1	0	2021-04-06
		Park	4	0	2021-03-12
		Platte	3	0	2021-04-29
		Sweetwater	1	0	2021-05-13
Eastern cottontail	Arkansas	Pulaski	0	2	N/A
	Colorado	Alamosa	1		2020-04-17
	Connecticut	Hartford	0	1	N/A
	Florida	Alachua	0	1	N/A
		Charlotte	0	1	N/A
		Collier	0	2	N/A
		Hillsborough	0	1	N/A
		Lee	0	5	N/A
		Martin	0	1	N/A
		Orange	0	1	N/A
		Palm Beach	0	2	N/A
		Pinellas	0	2	N/A
		Polk	0	1	N/A
		Sarasota	0	4	N/A
		Wakulla	0	1	N/A
	Georgia	Forsyth	0	2	N/A
		Hall	0	2	N/A
		Newton	0	1	N/A
		Putnam	0	1	N/A
		Walton	0	1	N/A
	Iowa	Cedar	0	1	N/A
		Clay	0	1	N/A
		Hamilton	0	1	N/A
		Marshall	0	1	N/A
		Polk	0	1	N/A
		Shelby	0	1	N/A
		Story	0	1	N/A
		Woodbury	0	1	N/A
	Illinois	Springfield	0	1	N/A
	Indiana	Elkhart	0	1	N/A
		Harrison	0	1	N/A
	Kansas	Coffey	0	1	N/A
		Lyon	0	2	N/A
		McPherson	0	2	N/A

		Sedgwick	0	1	N/A
		Thomas	0	1	N/A
	Kentucky	Allen	0	1	N/A
		Grayson	0	1	N/A
		Harlan	0	1	N/A
		Jefferson	0	1	N/A
		Oldham	0	1	N/A
		Robertson	0	1	N/A
	Louisiana	Grant	0	1	N/A
		LaSalle	0	5	N/A
		Natchitoches	0	1	N/A
		Rapides	0	3	N/A
	Massachusetts	Barnstable	0	1	N/A
		Essex	0	1	N/A
	Minnesota	Dakota	0	1	N/A
	Missouri	Boone	0	1	N/A
		Callaway	0	2	N/A
		Platte	0	1	N/A
		St. Louis	0	4	N/A
	North Carolina	Avery	0	1	N/A
		Carteret	0	1	N/A
		Cleveland	0	2	N/A
		Craven	0	2	N/A
		Cumberland	0	32	N/A
		Mecklenburg	0	13	N/A
		Orange	0	2	N/A
		Perquimans	0	1	N/A
		Randolph	0	1	N/A
		Richmond	0	19	N/A
		Rowan	0	1	N/A
		Scotland	0	4	N/A
		Stanly	0	2	N/A
		Transylvania	0	2	N/A
		Wake	0	1	N/A
	Nebraska	Box Butte	0	1	N/A
		Sarpy	0	1	N/A
	New Hampshire	Hillsborough	0	1	N/A
		Rockingham	0	2	N/A
	New Jersey	Burlington	0	1	N/A
	New York	Albany	0	2	N/A
		Bronx	0	1	N/A

		Erie	0	2	N/A
		Fulton	0	2	N/A
		Rensselaer	0	1	N/A
		Saratoga	0	1	N/A
		Suffolk	0	3	N/A
		Ulster	0	1	N/A
	Ohio	Cuyahoga	0	1	N/A
	Oregon	Benton	0	1	N/A
		Clackamas	0	7	N/A
		Deschutes	0	1	N/A
		Harney	1	0	2022-06-13
		Malheur	0	1	N/A
		Multnomah	0	4	N/A
		Washington	0	2	N/A
	South Carolina	Greenwood	0	1	N/A
	South Dakota	Pennington	1	1	2023-07-05
	Tennessee	Madison	0	1	N/A
	Texas	Angelina	0	2	N/A
		Brown	0	1	N/A
		Carson	0	1	N/A
		Denton	0	3	N/A
		Montague	0	1	N/A
		Trinity	0	1	N/A
	Virginia	Augusta	0	1	N/A
		Chesapeake	0	1	N/A
		Sussex	0	2	N/A
		Wythe	0	1	N/A
	Washington	Clark	0	1	N/A
		Island	1	0	2023-07-19
		King	0	1	N/A
		Thurston	0	1	N/A
		Walla Walla	0	1	N/A
	Wisconsin	Dane	0	3	N/A
		Eau Claire	0	1	N/A
		Waukesha	0	1	N/A
	West Virginia	Monongalia	0	1	N/A
	Wyoming	Albany	1	4	2020-12-16
		Goshen	4	1	2021-05-03
		Laramie	0	19	N/A
		Natrona	0	2	N/A
		Park	0	1	N/A

		Platte	0	1	N/A
		Washakie	0	1	N/A
Lepus sp.	Arizona	Maricopa	1	0	2021-05-18
	Colorado	Alamosa	1	0	2020-05-01
		Washington	1	0	2021-05-12
	New Mexico	Bernalillo	1	0	2022-12-08
	Texas	El Paso	1	0	2022-12-19
		Gillespie	1	0	2023-02-16
		Mason	1	0	2023-03-30
	Utah	Duchesne	1	0	2020-12-15
		San Juan	0	1	N/A
Marsh Rabbit	Florida	Martin	0	1	N/A
		Orange	0	7	N/A
		Pinellas	0	1	N/A
	North Carolina	Carteret	0	2	N/A
Mountain cottontail	Arizona	Apache	2	0	2020-05-01
		Coconino	2	1	2020-04-29
		Yavapai	0	1	N/A
	California	El Dorado	0	2	N/A
		Lassen	0	1	N/A
	Colorado	Moffat	1	0	2023-02-03
	Montana	Gallatin	0	1	N/A
		Yellowstone	1	1	2021-03-09
	Wyoming	Albany	0	1	N/A
		Park	0	1	N/A
New England cottontail	Maine	Cumberland	0	4	N/A
Pygmy rabbit	Nevada	Elko	1	0	2022-02-16
		Lander	6	0	2022-06-01
	Washington	Douglas	0	1	N/A
Riparian brush rabbit	California	Alameda	0	4	N/A
		Contra Costa	0	1	N/A
		Modesto	0	1	N/A
		San Joaquin	0	3	N/A
		Stanislaus	4	15	2022-05-20
Snowshoe hare	Minnesota	Itasca	0	1	N/A
	Montana	Missoula	0	1	N/A
	Oregon	Wallowa	0	1	N/A
	Pennsylvania	Monroe	0	1	N/A
	West Virginia	Tucker	0	1	N/A

	Wyoming	Sheridan	0	1	N/A
Swamp rabbit	Louisiana	Avoyelles	0	8	N/A
		Catahoula	0	1	N/A
		Franklin	0	2	N/A
Sylvilagus sp.	Alabama	Calhoun	0	1	N/A
	Arkansas	Fulton	0	1	N/A
	Arizona	Cochise	1	0	2020-04-08
		Maricopa	1	2	2021-05-18
		Navajo	0	1	N/A
	California	Los Angeles	0	3	N/A
		Orange	0	1	N/A
		San Bernardino	0	1	N/A
		San Diego	0	2	N/A
	Colorado	Adams	1	1	2021-05-12
		Alamosa	1	0	2023-06-12
		Arapahoe	0	2	N/A
		Broomfield	0	1	N/A
		Chaffee	1	0	2022-01-13
		Custer	2	0	2020-10-09
		Denver	1	0	2020-08-25
		Douglas	1	0	2022-03-11
		El Paso	4	0	2020-09-18
		Elbert	1	0	2020-09-29
		Garfield	1	0	2021-03-26
		Huerfano	1	0	2020-12-14
		La Plata	1	0	2020-10-23
		Larimer	9	0	2020-07-07
		Las Animas	1	0	2021-02-18
		Mesa	3	0	2020-08-18
		Montrose	1	0	2022-06-17
		Prowers	1	0	2020-05-07
		Pueblo	1	0	2020-05-22
		Rio Grande	1	0	2023-12-01
		Saguache	2	0	2021-03-26
		Weld	1	0	2021-06-02
	Connecticut	Hartford	0	1	N/A
		Litchfield	0	1	N/A
	Florida	Alachua	0	1	N/A
		Collier	0	1	N/A
		Lee	0	2	N/A

		Levy	0	1	N/A
		Manatee	0	1	N/A
		Osceola	0	1	N/A
		Palm Beach	0	3	N/A
		Pinellas	0	1	N/A
		Polk	0	1	N/A
		Sarasota	0	3	N/A
	Georgia	Bryan	0	1	N/A
		Cobb	0	1	N/A
		Columbia	0	1	N/A
		Dekalb	0	1	N/A
		Fayette	0	1	N/A
		Forsyth	0	1	N/A
		Fulton	0	1	N/A
		Gwinnet	0	2	N/A
		Hall	0	1	N/A
		Newton	0	1	N/A
	Iowa	Black Hawk	0	1	N/A
	Idaho	Ada	0	4	N/A
	Indiana	Bartholomew	0	1	N/A
	Kansas	Kearny	1	0	2023-05-01
		Leavenworth	0	1	N/A
		Riley	0	1	N/A
		Saline	0	1	N/A
	Kentucky	Marshall	0	1	N/A
	Massachusetts	Hampden	0	1	N/A
	Minnesota	Dakota	0	1	N/A
	Missouri	Adair	0	1	N/A
	Montana	Lewis and Clark	0	1	N/A
		Musselshell	1	0	2023-05-08
	North Carolina	Buncombe	0	2	N/A
		Halifax	0	1	N/A
		Mecklenburg	0	1	N/A
		Moore	0	1	N/A
		Warren	0	1	N/A
	Nebraska	Howard	0	1	N/A
	New Jersey	Atlantic	0	1	N/A
		Burlington	0	2	N/A
	New Mexico	Bernalillo	1	0	2020-06-30
		Sandoval	1	0	2023-07-03

	Nevada	Washoe	0	2	N/A
	New York	Suffolk	0	1	N/A
	Oklahoma	Adair	0	1	N/A
	Oregon	Benton	0	1	N/A
		Deschutes	1	1	2022-05-17
		Lane	0	2	N/A
		Linn	0	1	N/A
		Washington	0	2	N/A
	Pennsylvania	Northampton	0	1	N/A
	Rhode Island	Washington	0	1	N/A
	South Carolina	Greenwood	0	1	N/A
		Horry	0	1	N/A
		Kershaw	0	1	N/A
		Richland	0	1	N/A
		Sumter	0	1	N/A
	South Dakota	Beadle	0	2	N/A
		Custer	2	2	2024-03-06
		Jackson	0	1	N/A
	Tennessee	Blount	0	1	N/A
		Knox	0	1	N/A
	Texas	Brazoria	0	1	N/A
		Brewster	1	0	2020-06-11
		Dallas	0	1	N/A
		Ector	2	0	2023-03-16
		El Paso	2	0	2022-12-19
		Gillespie	0	1	N/A
		Jeff Davis	2	1	2020-05-21
		Lipscomb	1	0	2023-03-30
		Martin	1	0	2023-04-12
		Midland	2	0	2023-04-12
		Pecos	1	0	2023-02-24
		Potter	1	0	2020-06-22
		Randall	1	0	2023-05-04
		Reeves	1	0	2023-02-16
		Taylor	0	1	N/A
		Terrell	3	0	2023-02-07
		Terry	1	0	2023-04-12
		Trinity	0	1	N/A
		Ward	1	0	2020-05-21
	Utah	Grand	2	0	2021-01-20
		Millard	1	0	2021-02-15

		Salt Lake	0	1	N/A
		San Juan	2	0	2020-08-12
		Uintah	1	0	2020-12-04
		Washington	0	1	N/A
		Wayne	3	0	2020-07-20
	Washington	Snohomish	0	1	N/A
	West Virginia	Harrison	0	1	N/A
	Wyoming	Natrona	1	0	2021-04-19
		Park	3	0	2021-04-19
		Platte	0	1	N/A
White-tailed jackrabbit	Wyoming	Albany	0	1	N/A
		Laramie	0	2	N/A
